# High Cervical Spinal Cord Stimulation: A One Year Follow-Up Study on Motor and Non-Motor Functions in Parkinson’s Disease

**DOI:** 10.3390/brainsci9040078

**Published:** 2019-04-03

**Authors:** Paolo Mazzone, Fabio Viselli, Stefano Ferraina, Margherita Giamundo, Massimo Marano, Marco Paoloni, Francesco Masedu, Annamaria Capozzo, Eugenio Scarnati

**Affiliations:** 1 Functional Neurosurgery and DBS, Centro Chirurgico Toscano, 52100 Arezzo, Italy; 2 Department of Neurology, St John the Baptist Hospital, ACISMOM, 00148 Rome, Italy; f.viselli@acismom.it; 3 Department of Physiology and Pharmacology “Vittorio Erspamer” University of Rome La Sapienza, 00185 Rome, Italy; stefano.ferraina@uniroma1.it (S.F.); margherita.giamundo@uniroma1.it (M.G.); 4 Unit of Neurology, Neurophysiology and Neurobiology, Department of Medicine, Campus Bio-Medico University, 00128 Rome, Italy; m.marano@unicampus.it; 5 Department of Physical Medicine and Rehabilitation, University of Rome La Sapienza, 00185 Rome, Italy; marco.paoloni@uniroma1.it; 6 Department of Biotechnological and Applied Clinical Sciences (DISCAB), University of L’Aquila, 67100 L’Aquila, Italy; Francesco.Masedu@univaq.it (F.M.); capozzo@univaq.it (A.C.); scarnati@univaq.it (E.S.)

**Keywords:** cervical spinal cord stimulation, tonic stimulation, burst stimulation, Parkinson’s disease, gait, reaction time, Valsava maneuver

## Abstract

Background: The present study investigated the effectiveness of stimulation applied at cervical levels on pain and Parkinson’s disease (PD) symptoms using either tonic or burst stimulation mode. Methods: Tonic high cervical spinal cord stimulation (T-HCSCS) was applied on six PD patients suffering from low back pain and failed back surgery syndrome, while burst HCSCS (B-HCSCS) was applied in twelve PD patients to treat primarily motor deficits. Stimulation was applied percutaneously with quadripolar or octapolar electrodes. Clinical evaluation was assessed by the Unified Parkinson’s Disease Rating Scale (UPDRS) and the Hoehn and Yahr (H&Y) scale. Pain was evaluated by a visual analog scale. Evaluations of gait and of performance in a cognitive motor task were performed in some patients subjected to B-HCSCS. One patient who also suffered from severe autonomic cardiovascular dysfunction was investigated to evaluate the effectiveness of B-HCSCS on autonomic functions. Results: B-HCSCS was more effective and had more consistent effects than T-HCSCS in reducing pain. In addition, B-HCSCS improved UPDRS scores, including motor sub-items and tremor and H&Y score. Motor benefits appeared quickly after the beginning of B-HCSCS, in contrast to long latency improvements induced by T-HCSCS. A slight decrease of effectiveness was observed 12 months after implantation. B-HCSCS also improved gait and ability of patients to correctly perform a cognitive–motor task requiring inhibition of a prepared movement. Finally, B-HCSCS ameliorated autonomic control in the investigated patient. Conclusions: The results support a better usefulness of B-HCSCS compared to T-HCSCS in controlling pain and specific aspects of PD motor and non-motor deficits for at least one year.

## 1. Introduction

Spinal cord stimulation (SCS) is a well-established neuromodulatory therapy [1] for the treatment of neuropathic pain. Its rationale is based on the gate theory of pain, according to which, activation of myelinated large sensory fibers in the dorsal columns inhibits transmission of nociceptive signals carried by unmyelinated small fibers at the level of the dorsal horn [2,3,4,5].

Since its first application, SCS has undergone a profound evolution of tools, devices, and surgical procedures, and its field of application has been recently extended to various motor disorders and other pathologies [6,7,8].

Motor symptoms in Parkinson’s disease (PD) and PD animal models have been found to improve under SCS, and it has been reported that this treatment may also preserve nigrostriatal dopaminergic neurons from 6-hydroxydopamine-induced degeneration in rodents [9,10,11,12,13,14,15,16,17,18,19,20].

Most of the above clinical studies have been carried out by stimulating the spinal cord at thoracic levels. High cervical SCS (HCSCS) has been successfully introduced to manage pain from associated dermatomes (i.e., head, upper limbs, neck, and shoulders).

In 2010, we first described the acute clinical effects of tonic HCSCS (T-HCSCS) on motor functions in PD patients implanted at cervical levels to treat neuropathic pain [21]. That study did not provide evidence of significant acute improvements in all of the motor assessments performed. Thereafter, other authors reported heterogeneous and controversial effects on PD motor symptoms in investigations mainly based on thoracic SCS [13,22].

Because of these heterogeneous effects, different stimulation parameters and stimulation modalities have been applied in order to optimize stimulation, obtain the best clinical outcome, and minimize the paresthesias that often are associated with tonic stimulation. Among the stimulation modalities, burst stimulation (i.e., by delivering trains of high-frequency stimuli) has provided better pain relief without causing disabling paresthesias compared to tonic stimulation [23,24,25,26,27,28,29,30,31,32,33,34].

Given these results, in the present study, we investigated acute and long-term effects (i.e., after a 12-month follow-up) of HCSCS on motor and non-motor symptoms in PD patients and compared the effectiveness of T-HCSCS vs. burst HCSCS (B-HCSCS).

The study has been conducted mainly through conventional clinical evaluations. Some patients subjected to B-HCSCS were also assessed for gait and performance in a cognitive sensorimotor task. Finally, a patient affected by atypical parkinsonism who also suffered from severe autonomic disorder was studied to assess the effectiveness of B-HCSCS in improving autonomic cardiovascular control.

## 2. Materials and Methods

### 2.1. Ethics Approval

The study was approved by the local Ethics and human subject research committee, and a written informed consent was signed by all patients.

### 2.2. Patients

Eighteen patients suffering from PD or atypical parkinsonism who completed a one-year follow-up following implantation were enrolled in the study. Their demographic and clinical characteristics are reported in Table 1.

PD patients in Group I (6 males) were selected for T-HCSCS to treat mainly neuropathic low back pain, failed back surgery syndrome, or vascular pain. Camptocormia was present in two of these patients. They were aged 71.1 ± 7.3 years with disease duration of 17.1 ± 6.1 years.

PD patients in Group II (10 males and 2 females) underwent B-HCSCS to treat exclusively parkinsonian motor symptoms (i.e., tremor, rigidity, gait and posture disturbances). Camptocormia was present in one of these patients and pain in four patients. Criteria for including patients in Group II were the presence of other pathologies besides PD, ineligibility for Deep Brain Stimulation (DBS) of basal ganglia targets, ineffectiveness of subthalamic nucleus (STN) DBS (one patient), or decay of pedunculopontine nucleus DBS benefit (two patients). These patients were aged 65.6 ± 11.1 years and disease duration was 11.1 ± 5.3 years.

### 2.3. Surgical Procedure

Surgery was performed in the ASL RM2 Regional Center for Functional Neurosurgery and DBS, Operative Unit of Stereotactic and Functional Neurosurgery at the CTO Alesini Hospital in Rome in the period from April 2009 to December 2017. Local analgesia was employed in thirteen patients, and general anesthesia in five patients, depending on the patient’s conditions. Of the six patients in Group I, five were implanted with a quadripolar electrode (mod. 3487-A, Medtronic ND, Minneapolis, MI, USA) and one with an octapolar electrode (Pisces-Octad mod. 3898, Medtronic ND, Minneapolis, MI, USA). All patients in Group II were implanted with an MRI-compatible octapolar electrode (Octrode, St. Jude-Abbott, Austin, TX, USA).

**Figure 1:**
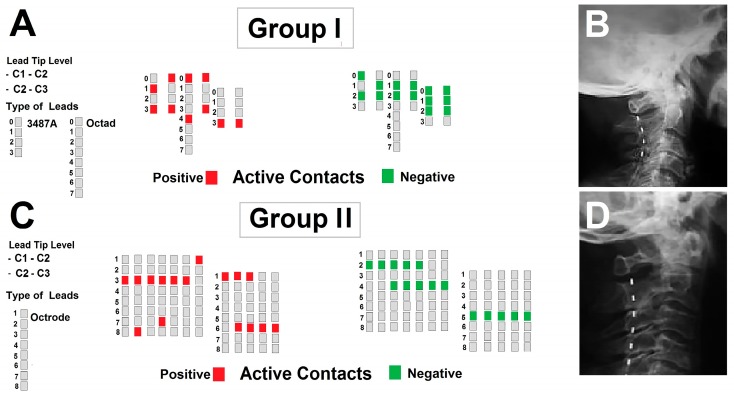
(**A,C**) Position of active contacts and type of electrode in Group I and Group II patients. (**B,D**) Representative examples of a quadripolar and octapolar electrode inserted at cervical level.

A pulse generator was applied to patients two weeks after electrode implantation. During these two weeks, the electrodes were externalized and connected to an external programmer to evaluate the acute effects of stimulation and ascertain that no unwanted effects occurred. For these aims, a Synergy Versitrel device (Medtronic, Minneapolis, MI, USA) was used in Group I patients, while a Prodigy current constant device (St. Jude Medical, St.Paul, MI, USA) was used in Group II patients. During external temporary stimulation, the acute effects of both T-HCSCS and B-HCSCS were assessed in Group II patients in successive sessions, and the current threshold for the appearance of paresthesias was established in each patient. The most useful pairs of stimulation contacts and the correct positioning of the electrode, as postoperatively verified by X-ray, are shown in Figure 1.

### 2.4. Stimulation Parameters and Clinical Evaluations

The pair of stimulation contacts providing the best clinical outcome was searched in each patient, and optimal stimulation parameters (frequency, pulse duration, current amplitude, or intensity) were determined (Table 2). Adjustments of current levels were made in the course of the follow-up to assure the constancy of clinical outcome. Patients in Group I had the possibility to modify by themselves one of the three stimulation parameters (i.e., frequency or pulse duration or current intensity), within a range previously established by physicians, as they felt a decline of the efficacy of stimulation. Patients in Group II also had the possibility to modify the pair of active contacts according to preprogrammed combinations. The number of adjustments made by both physicians and patients was considered an index of the stability of stimulation efficacy. Pain was evaluated through a visual analog scale (VAS). Clinical examination was performed preoperatively, 2 days after beginning of stimulation (acute evaluation) and 3, 6, and 12 months later. For this aim, the Unified Parkinson’s Disease Scale (UPDRS) III, sub-item 16 (tremor), sub-items 27–31 (arising from chair, posture, gait, postural stability, and body bradykinesia and hypokinesia), the Hoehn and Yahr (H&Y) scale, and the VAS were evaluated in the conditions of stimulation-OFF/drug-ON and stimulation-ON/drug-ON. The condition of stimulation-OFF/drug-ON was investigated after switching off stimulation for 48 h. Drug therapy for PD was never suspended for ethical reasons, and the daily dose of L-Dopa (mg/day) was modified in the course of the follow-up according to patients’ responsiveness to stimulation.

Patients in Group II underwent the same general procedures as Group I patients. They began to receive stimulation 2 days after electrode implantation. Stimulation was applied in the tonic modality in the first two days to establish the current threshold for the appearance of paresthesias. Then, stimulation was switched to the burst modality, i.e., at a burst rate of 40 Hz, intraburst rate of 500 Hz, pulse width of 1000 µs, and current intensity of 0.5 ± 0.2 mA. When starting burst stimulation, the current intensity was set at 80% of the threshold for the appearance of paresthesias. During the follow-up, the current intensity was reduced up to 60% of the threshold for paresthesias in most patients.

### 2.5. Instrumental Evaluation of Gait

Instrumental analysis of gait was carried out in eight patients (four in Group I and four in Group II) who manifested willingness to undergo the procedure for gait analysis. This was done 6 months after beginning of stimulation. Spatiotemporal parameters of gait were investigated in conditions of either stimulation OFF or stimulation ON, and always under medication, according to methods described previously [35,36]. Four height-, weight-, and age-matched male subjects served as controls.

### 2.6. Cognitive Motor Task

Three patients in Group II who manifested willingness to undergo a stop-signal (SS) behavioral task were assessed for their motor abilities in a motor-related cognitive task in the first days following surgery when the electrodes were still externalized. These patients were selected since, in addition to motor symptoms, they clearly showed clinical signs of slowing of motor initiation during neurological examination. The SS task has been widely used to evaluate the involvement of cortical and subcortical brain regions on the cognitive control of movement in normal subjects and patients [37,38,39,40]. The inhibitory control of acts and thoughts is part of the executive functions managed by the prefrontal cortex in cooperation with many other cortical and subcortical regions, primarily the basal ganglia [41].

During the experiments, the patients were seated in a dimly lit room, with their eyes 45 cm from a standard PC monitor (cathode-ray tube not interlaced, rate 85 Hz). The presentation of visual stimuli and data acquisition w under the control of the freeware Psychtoolbox software ver. 3.0 (psychtoolbox.org) operating under MatLab (www.matworks.com). A joystick, aligned to the body midline, was connected to a Universal Serial Bus computer port and used to measure motor parameters.

**Figure 2:**
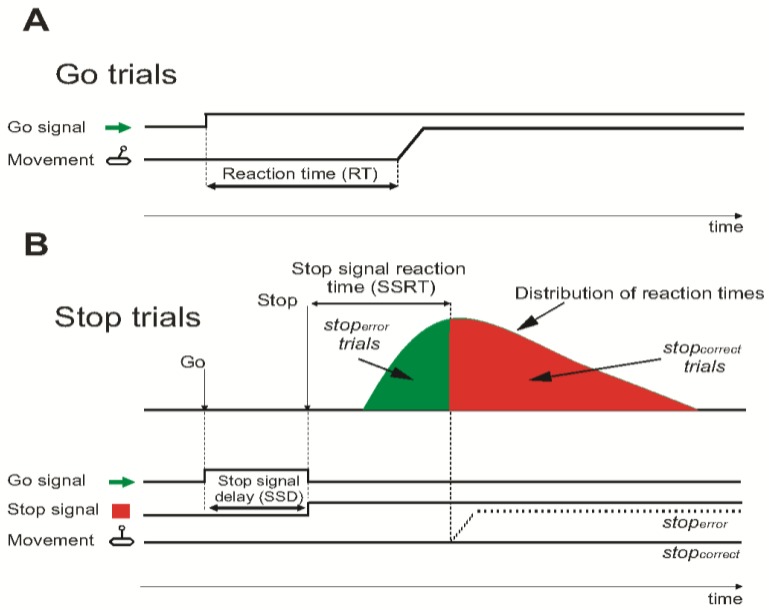
Schematic representation of the cognitive–motor task. (**A**) In Go trials, the subjects had to respond by moving a joystick at the presentation of a green arrow to generate the reaction time (RT). (**B**) In stop trials, they had to suppress the ongoing movement if a red square was presented in the interval within the reaction time window of the Go trial. The interval between presentation of the stop signal and suppression of movement represented the stop signal reaction time (SSRT).

In the task (Figure 2), the subjects were instructed to respond in most of the trials with a directed motor action on the joystick in the direction indicated by the Go signal (arrow). In selected trials, a Stop signal (SS, red square) replaced the Go signal during the reaction time (RT) after a variable stop signal delay (SSD) instruction in order to cancel the programmed movement. Trials with successfully suppressed movements were classified as stop-correct, while trials with movements executed after a SS presentation were classified as stop-error.

A staircase procedure was used to select the SSD in each stop trial. The SSD was increased by 50 ms after each stop-correct trial and decreased by 50 ms after each stop-error trial. The procedure automatically adapted the SSD duration to subject performance to obtain a session probability of stop-error that approximates 0.5, and that was similar between patients. The RT distribution and the relationship between the different SSDs used and the corresponding probability of success allowed us to obtain an estimate of the efficiency of the inhibitory process (SS reaction time, SSRT) [41].

All these task-specific, behaviorally relevant variables were studied in the phase in which B-HCSCS was applied through the externalized electrode, i.e., before the final procedure to connect the electrode to the subcutaneous stimulator. The task was investigated under stimulation in the morning (ON condition) and 30–40 min after switching off the stimulator in the afternoon (OFF condition). Blocks of 100 trials were employed (3 blocks per patient in ON condition and 3 in OFF condition, total trials: 300 in ON condition and 300 in OFF condition; percentage: Stop 30% and Go 70%). All patients showed motivation and fully collaborated during the experimental sessions. Three age-matched subjects served as controls.

### 2.7. Autonomic Function Evaluation

One patient in Group II who was affected by atypical parkinsonism (multiple system atrophy) and showed severe cardiovascular autonomic dysfunction was investigated to assess to what extent B-HCSCS could improve autonomic responses. This was done according to principles, methods, and materials described elsewhere [42,43,44].

In brief, the sympathetic nervous system was assessed by recording blood pressure responses to the head-up tilt test (HUTT), the Valsalva maneuver, and the handgrip test. The parasympathetic cardiovagal function was assessed by recording heart rate variation during deep breathing and Valsava maneuver.

### 2.8. Statistic Analysis

Clinical data were evaluated using Friedman nonparametric ANOVAs, and the Wilcoxon test was used for pairwise comparisons. Gait and cognitive data were analyzed using a nonparametric ANOVA design, and pairwise comparisons were performed using the Mann–Whitney test, with Bonferroni’s adjusted *p*-values if required. Type I error was set at *p* < 0.05. The Statistica ver. 8.0 software package (Statsoft, Tulsa, OK, USA) was used. Values are reported as means ± SD.

## 3. Results

No complications occurred during electrode implantation and in the follow-up period. The electrode tip was located at C2 level in sixteen patients and at C2–C3 in two patients (Figure 1)

### 3.1. T-HCSCS (Group I Patients)

The best clinical outcome was obtained by delivering stimuli at rate of 150 ± 27.2 Hz, pulse width 93.3 ± 58.5 µs, and voltage of 3.0 ± 0.9 V. The most useful contact pair was contact 2 as the cathode and contact 3 as the anode. These patients needed to vary the electrical parameters often to maintain the efficacy of stimulation, and a mean of 17.6 ± 5.7 modifications were made every three months in the follow-up period.

**Figure 3:**
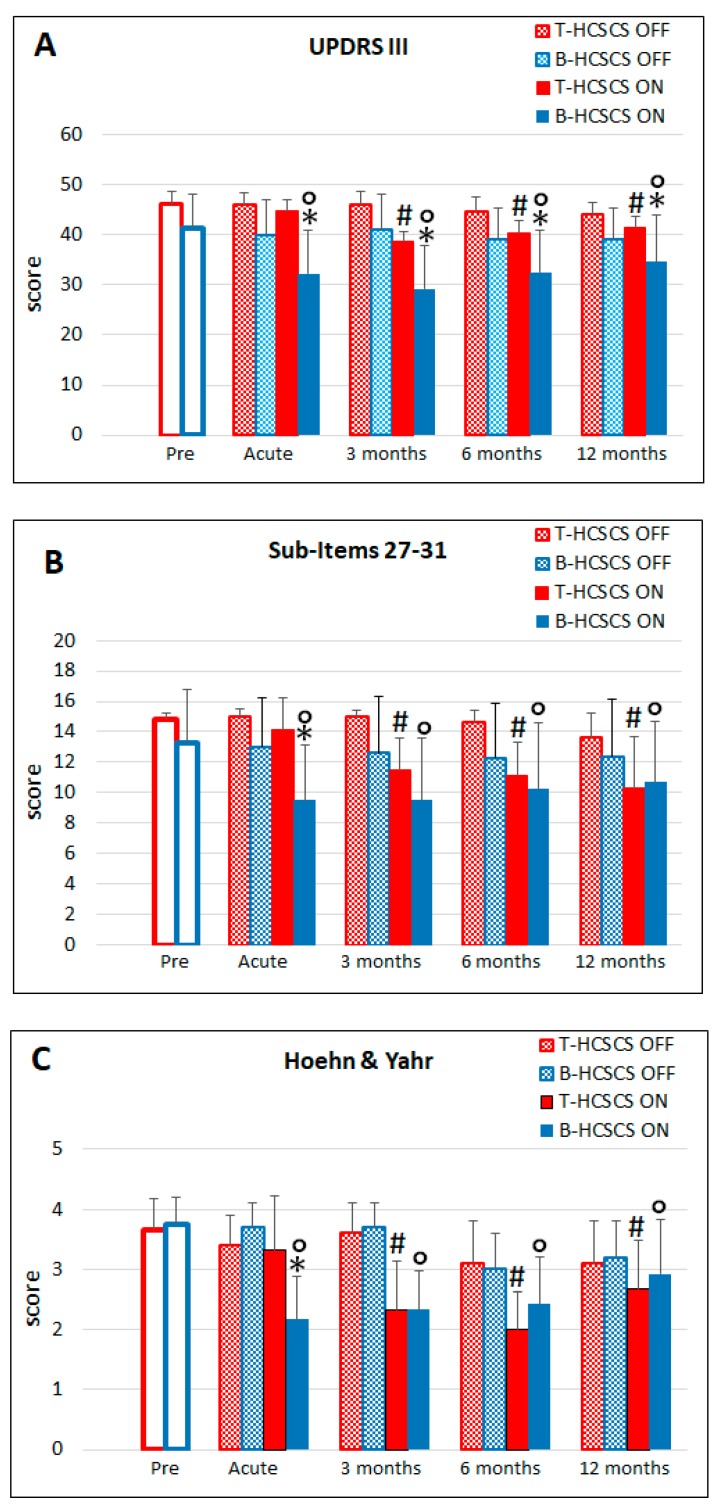
(**A**) Tonic stimulation (**red**) UPDRS scores at 3, 6, and 12 months differed significantly compared to presurgery, stimulation OFF, and acute stimulation values (# *p* < 0.001). Burst stimulation (**blue**) improved UPDRS scores significantly after acute stimulation as well as at 3, 6, and 12 months (° *p* < 0.001) compared to presurgery and stimulation OFF values. Burst stimulation was more effective than tonic stimulation at all the timepoints considered (* *p* < 0.001). (**B**) 27–31 motor sub-items evaluations at 3, 6, and 12 months differed significantly during tonic stimulation compared to presurgery, stimulation OFF, and acute stimulation values (# *p* < 0.05). Burst stimulation significantly improved motor sub-items comparing acute, stimulation OFF and 3, 6, and 12 months to presurgery value (° *p* < 0.001). Burst stimulation was significantly more effective than tonic stimulation in the acute condition (* *p* < 0.05). (**C**) Hoehn and Yahr evaluations at 3, 6, and 12 months differed significantly during tonic stimulation compared to presurgery, stimulation OFF, and acute stimulation values (# *p* < 0.001). Burst stimulation significantly improved Hoehn and Yahr score comparing acute, 3, 6, and 12 months to presurgery and stimulation OFF values (° *p* < 0.001). Burst stimulation was significantly more effective than tonic stimulation in the acute condition (* *p* < 0.05). Statistics were assessed by Friedman tests.

**Figure 4:**
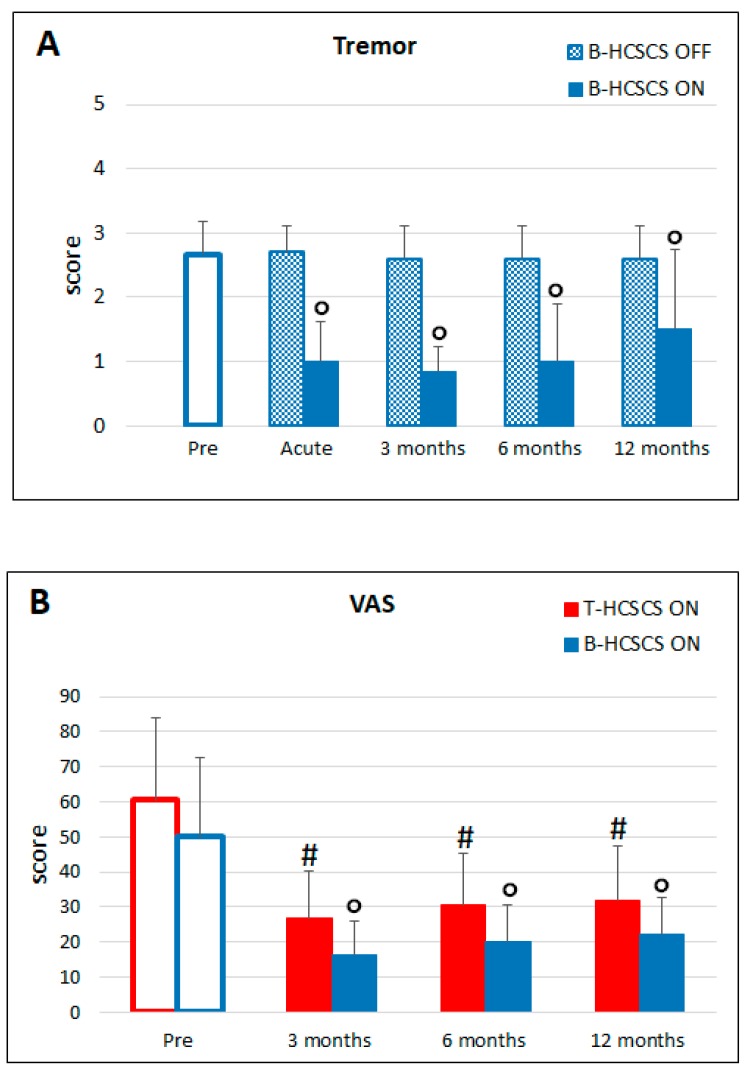
(**A**) Tremor was significantly reduced during burst stimulation at all time points compared to presurgery and stimulation OFF values (° *p* < 0.05). (**B**) Pain at 3, 6, and 12 months poststimulation was significantly reduced by both types of stimulation compared to the presurgery condition (#° *p* < 0.05). Statistics were assessed by Friedman tests.

As shown in Figure 3 and Figure 4, stimulation required three months of application to induce significant improvement in UPDRS evaluations, including sub-items 27–31, except in one patient, in whom a prompt improvement of motor disabilities was observed switching on stimulation the day after implantation. This benefit was still present at 12 months post-implantation. In agreement with this result, the daily dose of L-Dopa could be reduced from the preoperative value of 1333.3 ± 471.9 mg to 1083.3 ± 264.0 mg at 12 months.

The H&Y score also required three months of stimulation to show a reduction. This benefit declined at 12 months but remained at significant levels compared to the preoperative values. Concerning pain, the VAS score showed a significant reduction up to the ninth month of stimulation and, therefore, the administration of analgesics could be reduced. This effect lasted until 12 months post-implantation, when pain returned to preoperative level.

**Figure 5:**
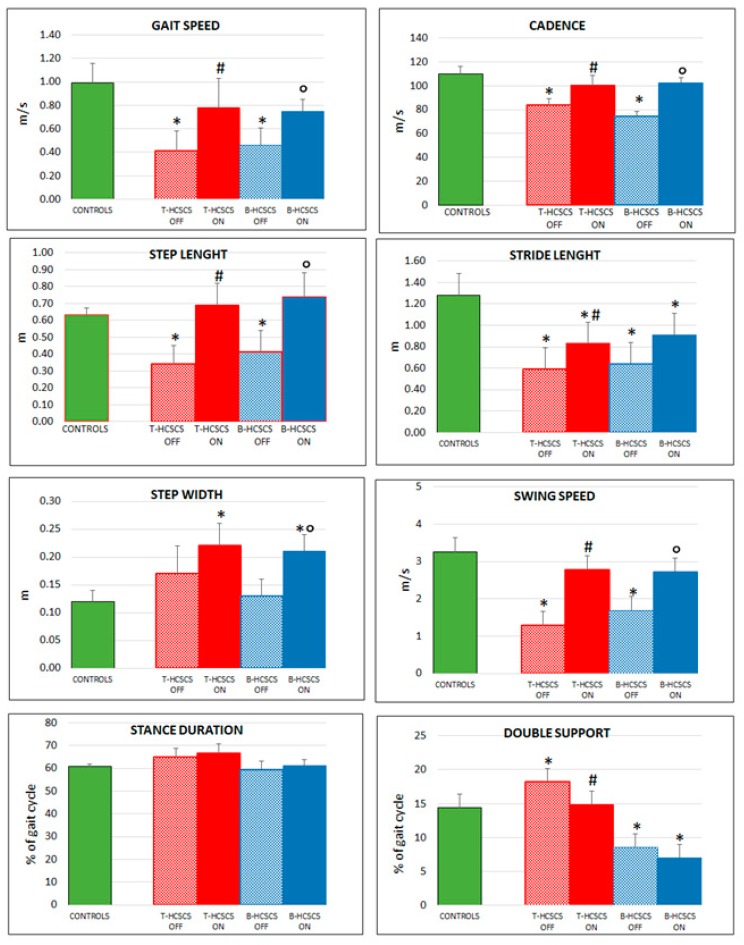
Improvement of spatiotemporal gait parameters (gait speed, cadence, step length, stride length, step width, swing speed, stance duration, double support) in the two groups of patients investigated at six months under T-HCSCS (**red**) or B-HCSCS (**blue**). * *p* < 0.05: Control vs. T-OFF and B-OFF; # T-OFF vs. T-ON; ° B-OFF vs. B-ON (except cadence: *p* < 0.001) and stance duration (not significant). Statistics were assessed by Mann–Whitney tests.

The motor improvements that could be appreciated upon clinical examination were also supported by the results of instrumental evaluation as many of the of gait parameters, such as cadence, step length, and stride length, which significantly ameliorated during stimulation (Figure 5).

### 3.2. B-HCSCS (Group II Patients)

The best clinical motor outcome was obtained by delivering bursts at 40 Hz, with each burst composed of five 1 ms pulses delivered at 500 Hz and at 0.5 ± 0.2 mA current intensity.

Among the combinations of active contacts that could be selected in the octapolar electrode, contacts two, four, and five used as the cathode and contacts three and six as the anode gave the best clinical result. The number of adjustments of the electrical parameters that these patients required every three months was significantly lower than in Group I patients (3.9 ± 0.9 vs. 17.6 ± 5.6, *p* < 0.05), and the intensity of the stimulation current could be reduced up to 60% of the level that produced paresthesias.

As shown in Figure 3 and Figure 4, efficacy of stimulation developed soon after the beginning of stimulation, lasted until the end of the follow-up, and was more effective than T-HCSCS. The higher efficacy of B-HCSCS vs. T-HCSCS was still present at 12 months, although at a lesser extent compared to previous UPDRS evaluations.

Sub-item 27–31 scores showed a significant improvement in gait and motor abilities that was maintained at 12 months post-implantation. This effect was more effective compared to the effect of T-HCSCS, but from the sixth month onward, there was no difference between the two stimulation modalities.

The H&Y score already decreased at the first evaluation after beginning of stimulation and remained at values similar to those recorded in T-HCSCS during the follow-up, to ultimately decline at 12 months.

Tremor decreased significantly after beginning of stimulation and was still significantly reduced at 12 months, although to a lesser extent. In line with the improvement of motor disabilities, the daily dose of L-Dopa could be reduced from the preoperative value of 835.0 ± 310.1 mg to 730 ± 273.7 mg at 12 months.

The B-HCSCS effect did not improve camptocormia in the patients that manifested this postural deformity. Concerning pain, it was significantly and consistently reduced throughout the follow-up (not shown); consequently, a lower number of analgesic drugs were administered than in the preoperative condition. The maximal efficacy of B-HCSCS in reducing pain compared to T-HCSCS was reached at 12 months post-implantation.

As shown in Figure 5, the instrumental spatiotemporal analysis of gait provided objective data supporting the improvement of gait suggested by the clinical evaluation of UPDRS 27–31 motor sub-items. In general, these patients showed better basic parameters, likely because of their younger age compared to Group I patients. As it occurred with tonic stimulation, gait parameters improved, although never reaching values of age-matched healthy controls. Cadence, step length, and stride length increased significantly, while gait speed, step width, speed swing, percent of stance, and percent of double support improved but did not reach levels of significance compared to prestimulation values.

### 3.3. Cognitive–Motor Task

All subjects performed in the stop-signal task as required, suggesting that they were able to adequately follow the provided instructions. The behavioral adhesion to the race model previsions was controlled before computing the SSRT through the integration method [45]. For this aim, we controlled that the mean stop-error RT in each subject was significantly lower than the mean Go trials RT (paired-t test). We also controlled for an increase of the stop-error RT with SSD duration. Regression analysis detected across subjects showed a significant linear increase of the stop-error RT with the SSD (not shown).

**Figure 6:**
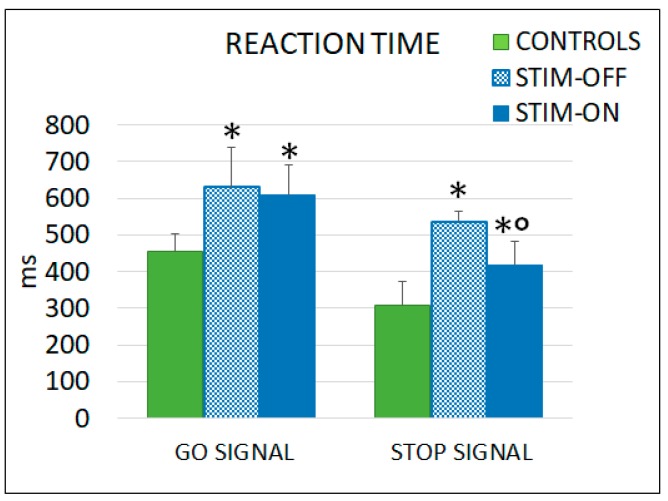
In patients, burst stimulation reduced the duration of the SSRT, while the RT duration was unaffected. Go signal: * *p* < 0.05 controls vs. OFF and vs. ON; Stop signal: * *p* < 0.05 controls vs. OFF and vs. ON, ° *p* < 0.05 OFF vs. ON. Statistics were assessed by Mann–Whitney tests.

As shown in Figure 6, RT and SSRT were significantly higher in PD patients compared to controls. In patients, spinal stimulation reduced only the duration of the SSRT, while it did not affect the RT.

### 3.4. Effects on Vegetative Functions

Table 3 shows the effects induced by HCSCS stimulation on the patient affected by severe autonomic dysfunction. In stimulation OFF, the patient showed orthostatic hypotension, absence of the pressure overshoot that occurs in phase IV of the Valsalva maneuver, and a reduced increase of the diastolic pressure during the handgrip test. When stimulation was applied, the vegetative responses improved, except the cardiovagal response to the deep breathing test.

## 4. Discussion

Stimulation of the dorsal columns of the spinal cord at thoracic levels has been initially introduced to control neuropathic pain [3,4,11]. Given its effectiveness, stimulation has also been applied at cervical level for treating head, upper limbs, neck, and shoulder pain conditions. However, when applied in PD patients and PD animal models, spinal stimulation was also effective in reducing motor disturbances typical of this neurodegenerative disorder [10,15,18,19,22,46,47].

In our former experience [21], short-term effects of T-HCSCS, i.e., when stimulation was continuously applied to the upper cervical level 48 h after electrode implantation, were represented by reduction of pain, whereas akinesia and locomotion were not significantly improved.

By contrast, long term effects of T-HCSCS showed that PD motor symptoms could be ameliorated and that these effects declined after several months of stimulation. Similar results were also reported in the literature [22].

The reduction of motor improvement varied from patient to patient, and frequent adjustments of stimulation parameters were required to maintain stimulation effectiveness. When an increase of current intensity was requested, patients suffered from paresthesias to the point that no further change could be made. The most parsimonious explanation of the loss of effectiveness of tonic stimulation over time is that adaptation to continuously applied stimuli developed.

Given the above data, in the present study, we applied B-HCSCS in a larger group of PD patients who were not eligible for STN or globus pallidus DBS, or in which DBS had not provided satisfactory results, and we compared the effects of T-HCSCS with those induced by B-HCSCS. The latter mode of stimulation has been recently introduced in our practice in treating pain syndromes and revealed better effectiveness than T-HCSCS in providing a long-lasting relief and a better quality of life (QoL).

While confirming our prior results, the present study revealed that there are important qualitative and quantitative differences in the effects induced by the two stimulating modalities. B-HCSCS-treated patients improved in UPDRS scores, including motor sub-items 27–31, within a few minutes after stimulation onset, and tremor was also reduced. These results were supported by improvements in gait performance and psychomotor abilities in some patients whom it was possible to enroll for instrumental analysis of gait and psychomotor capacity. The limits of the gait analysis and evaluation of psychomotor abilities that we present are due to the small cohort of patients enrolled and by the single evaluation that it has been possible to perform only at 5–6 months following stimulation. In spite of these limits, the collected results are worth consideration, given the overall results and conclusion of this study.

When delivering B-HCSCS, fewer changes to stimulation parameters were required throughout the follow-up than those required when using T-HCSCS. In addition, the current intensity could be reduced up to 60% of the threshold for paresthesias. These two factors, together with the lower amount of L-Dopa that was possible to administer, decreased the H&Y score and improved the patients’ QoL. It should also be noted that the ability that patients had to change the stimulation current on their own when they felt that stimulation began to be ineffective should have minimized the issue of adaptation to stimuli, increasing the psychological confidence of the patient in the method and favoring a consistent clinical improvement over the follow-up period.

Concerning the mechanism of action, the different clinical effects on pain and motor disabilities induced by the two types of stimulation and the different time-course of their effects provide further support to the hypothesis that B-HCSCS and T-SCSCS may act through different spinal and supraspinal mechanisms [23,26,48], neurotransmitters, and neuroactive substances [48,49].

The results that we have obtained with tonic stimulation are fully in agreement with those reported by Pinto de Souza and co-workers, who found 50% to 65% improvement in gait measurements and 35% to 40% in UPDRS III and QoL scores after stimulating the spinal cord at thoracic levels in four PD patients resistant to STN DBS [18]. In addition, our study provides clinical and instrumental data supporting better and long-lasting effectiveness of burst stimulation compared to tonic stimulation of the upper segments of the cervical cord. Such benefits were also observed in patients affected by atypical parkinsonisms.

The positive effects that we report by applying stimulation at high cervical levels, i.e., at a level closer to brainstem structures compared to the more distant thoracic levels, suggest that cervical stimulation, in addition to modulating the activity of dorsal column and dorsal horn elements involved in pain control, may also act by modulating the activity of motor and vegetative brainstem nuclei.

In this regard, it should be also emphasized that the most caudal contact of the stimulating electrode was used as the cathode of the active contacts pair, while the anode was chosen among the remaining contacts. This means that the current flow was always directed toward structures located in the upper cervical segments and overlying brainstem.

Further support for this assumption stems from the improvement of autonomic control on cardiovascular functions induced by B-HCSCS in the patient suffering from atypical parkinsonism, who also suffered from a severe autonomic cardiovascular dysfunction. Despite the limitations that a single case study may have, the results from this patient are in agreement with the possible involvement of brainstem nuclei in the mediation of HCSCS effects. Indeed, the brainstem, in addition to the dorsal column nuclei, includes both nuclei controlling autonomic functions and motor nuclei, such as the cuneiform and the pedunculopontine (PPN) nuclei. These latter nuclei are involved in both motor and non-motor functions, and their DBS has been shown to improve gait and axial deficits in PD [35,36,50,51,52]. Another possibility is that stimulation may activate gamma oscillations in the PPN (~40 Hz), thus generating bottom–up gamma-inducing normal levels of activity [53].

B-HCSCS was also effective in improving the inhibitory control of an ongoing movement rather than reaction to an imperative signal for movement initiation. The reasons of this effect remain to be investigated, but, according to data in the literature, the frontal lobe [54,55,56,57,58] and the hyperdirect pathway, i.e., the cortico–subthalamic–pallidal pathway that mediates fast inhibition of movement [59,60], might play a role in such an effect.

If one looks at the effects of acute application of B-HCSCS and DBS of basal ganglia nuclei, it appears that both these effects share a rapid onset. Assuming that B-HCSCS may influence basal ganglia machinery in the brainstem, it is reasonable to hypothesize that B-HCSCS and basal ganglia DBS may share some mechanisms [61,62,63,64,65,66].

Conversely, T-HCSCS, which requires a longer application time to be effective, might require additional changes in the nervous system that establish slowly, perhaps requiring long-latency action of neurotransmitters and neuroactive substances [67,68,69].

## 5. Conclusions

B-HCSCS appears to be more effective than T-HCSCS in improving pain as well as motor disabilities and tremor in PD and atypical parkinsonisms at least after one year of application. These improvements may be associated with better cognitive performance and amelioration of autonomic control. The benefits of B-HCSCS appear to develop quickly and persist for a longer time compared to those induced by T-HCSCS. In addition, using the burst mode of stimulation, a better control of paresthesias may be obtained by reducing stimulation current and, at the same time, a better QoL may be provided to patients in reducing the daily L-Dopa administration.

Further investigation in larger and homogeneous cohorts of patients and controls will help in the future to elucidate the functional consequences of B-HCSCS and their long-lasting time course.

## Figures and Tables

**Table 1:** Demographic and clinical characteristics of patients.

	Patients	Age	Gender	Disease	Duration (years)	L-Dopa Pre-Op (mg/day)	L-Dopa Post-Op (mg/day)	HCSCS Early Indication
**Group I**	1	76	M	LBP + IPD	18	1800	1300	Pain
	2	79	M	VP + AP *	20	600 °	600	Pain
	3	58	M	VPD	16	1000	1000	PD
	4	74	M	VP + VPD	27	1500	1200	Pain
	5	71	M	FBSS + IPD *	9	1300	1100	Pain
	6	69	M	LBP + IPD	13	1800	1300	Pain
**Mean ± SD**		71.1 ± 7.3			17.1 ± 6.1	1334 ± 472	1083.3 ± 263.9	
**Group II**	7	72	M	AP	5	(-)	(-)	AP
	8	48	M	VPD	11	850	850	PD
	9	65	F	IPD	5	500	350	PD
	10	61	M	VPD	11	1200	750	PD
	11	73	M	VPD	16	800	800	PD
	12	73	M	VPD	14	1000	750	PD
	13	76	M	VPD	9	650	650	PD
	14	81	M	VP + AP *	20	600	600	PD/Pain
	15	64	F	VPD	8	350	350	PD
	16	63	M	VPD	20	1200	1200	PD
	17	43	M	VPD	9	1200	1000	PD
	18	69	M	AP	5	(-)	(-)	AP
**Mean ± SD**		65.6 ± 11.1			11.1 ± 5.3	835 ± 310 (§)	730 ± 263.7	

IPD: Idiopathic Parkinson’s disease; VPD: Vascular Parkinson’s disease; AP: Atypical parkinsonism; LBP: Low back pain; VP: Diffuse vertebral pain; FBSS: Failed back surgery syndrome; PD: Parkinson’s disease; (*) camptocormia; (°) intense dystonic–dyskinetic effect of drugs; (-) nonresponsive to L-Dopa; (§) non-dyskinetic dosage.

**Table 2:** Stimulation parameters.

Patients	Hz ON	Hz OFF	Pulse Width (µs)	Intensity	Electrical Changes Every 3 Months	Improvement
**Group I**						
1	185	-	80	2.5 V	9	delayed
2	130	-	60	3.6 V	16	delayed
3	130	-	90	4.0 V	15	acute
4	185	-	210	1.3 V	26	delayed
5	135	-	60	3.5 V	21	delayed
6	135	-	60	3.5 V	19	delayed
**Mean ± SD**	150.0 ± 27.2		93.3 ± 58.5	3.0 ± 0.9	17.6 ± 5.7	
**Group II**						
7	500	40	1000	0.9 mAmp	4	acute
(*) 8	500	40	1000	0.6 mAmp	5	acute
(*) 9	500	40	1000	0.7 mAmp	4	acute
10	500	40	1000	0.9 mAmp	4	acute
(*) 11	500	40	1000	0.7 mAmp	4	acute
12	250	40	1000	0.6 mAmp	2	acute
13	500	40	1000	0.7 mAmp	4	acute
(*) 14	500	40	1000	0.3 mAmp	5	acute
15	500	40	1000	0.2 mAmp	3	acute
(*) 16	500	40	1000	0.5 mAmp	5	acute
17	500	40	1000	0.4 mAmp	3	acute
(*) 18	500	40	1000	0.2 mAmp	4	acute
**Mean ± SD**	479.2 ± 72.2	40	1000	0.5 ± 0.2	3.9 ± 0.9	

(*) Current intensity in these patients could be reduced up to 60% the threshold for paresthesias.

**Table 3:** Effects of burst high cervical spinal cord stimulation (B-HCSCS) on the patient suffering autonomic nervous system dysfunctions.

	Stimulation OFF	Stimulation ON
**Head-up tilt test**		
∆ SBP (mmHg)	−41	−17
∆ DBP (mmHg)	−13	16
∆ HR (bpm)	11	22
**VALSAVA**		
OV mmHg	Absent	4
VR	1.41	1.21
**Hand grip test**		
∆ DBP (mmHg)	3	15
**Deep–Breathing test**		
I-E difference (bpm)	11	16

∆ SBP, variation in systolic blood pressure; ∆ DBP, variation in diastolic blood pressure; ∆ HR, variation in heart rate; OV, pressure overshoot in Valsava Manouver; VR, Valsava ratio; I-E difference in heart beats per minute during inspiration–expiration in deep breathing.

## References

[1] Shealy C.N., Mortimer J.T., Reswick J.B. (1967). Electrical inhibition of pain by stimulation of the dorsal columns: Preliminary clinical report. Anesth. Analg..

[2] Gildenberg P.L. (2003). History of neuroaugmentative procedures. Neurosurg. Clin. N. Am..

[3] Melzack R., Wall P.D. (1965). Pain mechanisms: A new theory. Science.

[4] Melzack R., Wall P.D. (1970). Evolution of pain theories. Int. Anesthesiol. Clin..

[5] Ropero Pelaez F.J., Taniguchi S. (2016). The Gate Theory of Pain Revisited: Modeling Different Pain Conditions with a Parsimonious Neurocomputational Model. Neural Plast..

[6] Grill W.M., Craggs M.D., Foreman R.D., Ludlow C.L., Buller J.L. (2001). Emerging clinical applications of electrical stimulation: Opportunities for restoration of function. J. Rehabil. Res. Dev..

[7] Kowalski K.E., Romaniuk J.R., Kowalski T., DiMarco A.F. (2017). Effects of expiratory muscle activation via high-frequency spinal cord stimulation. J. Appl. Physiol (1985).

[8] Meier K. (2014). Spinal cord stimulation: Background and clinical application. Scand. J. Pain.

[9] Fuentes R., Petersson P., Siesser W.B., Caron M.G., Nicolelis M.A. (2009). Spinal cord stimulation restores locomotion in animal models of Parkinson’s disease. Science.

[10] Fuentes R., Petersson P., Nicolelis M.A. (2010). Restoration of locomotive function in Parkinson’s disease by spinal cord stimulation: Mechanistic approach. Eur. J. Neurosci..

[11] Mendell L.M. (2014). Constructing and deconstructing the gate theory of pain. Pain.

[12] Shinko A., Agari T., Kameda M., Yasuhara T., Kondo A., Tayra J.T., Sato K., Sasaki T., Sasada S., Takeuchi H. (2014). Spinal cord stimulation exerts neuroprotective effects against experimental Parkinson’s disease. PLoS ONE.

[13] Yadav A.P., Nicolelis M.A.L. (2017). Electrical stimulation of the dorsal columns of the spinal cord for Parkinson’s disease. Mov Disord..

[14] Yadav A.P., Fuentes R., Zhang H., Vinholo T., Wang C.H., Freire M.A., Nicolelis M.A. (2014). Chronic spinal cord electrical stimulation protects against 6-hydroxydopamine lesions. Sci. Rep..

[15] Agari T., Date I. (2012). Spinal cord stimulation for the treatment of abnormal posture and gait disorder in patients with Parkinson’s disease. Neurol. Med. Chir (Tokyo).

[16] Hassan S., Amer S., Alwaki A., Elborno A. (2013). A patient with Parkinson’s disease benefits from spinal cord stimulation. J. Clin. Neurosci..

[17] de Lima-Pardini A.C., Coelho D.B., Souza C.P., Souza C.O., Ghilardi M.G.D.S., Garcia T., Voos M., Milosevic M., Hamani C., Teixeira L.A. (2018). Effects of spinal cord stimulation on postural control in Parkinson’s disease patients with freezing of gait. Elife.

[18] Pinto de S.C., Hamani C., Oliveira S.C., Lopez Contreras W.O., Dos Santos Ghilardi M.G., Cury R.G., Reis B.E., Jacobsen T.M., Talamoni F.E. (2017). Spinal cord stimulation improves gait in patients with Parkinson’s disease previously treated with deep brain stimulation. Mov. Disord..

[19] Fenelon G., Goujon C., Gurruchaga J.M., Cesaro P., Jarraya B., Palfi S., Lefaucheur J.P. (2012). Spinal cord stimulation for chronic pain improved motor function in a patient with Parkinson’s disease. Parkinsonism Relat. Disord..

[20] Thiriez C., Gurruchaga J.M., Goujon C., Fenelon G., Palfi S. (2014). Spinal stimulation for movement disorders. Neurotherapeutics.

[21] Thevathasan W., Mazzone P., Jha A., Djamshidian A., Dileone M., Di Lazzaro V., Brown P. (2010). Spinal cord stimulation failed to relieve akinesia or restore locomotion in Parkinson disease. Neurology.

[22] de Andrade E.M., Ghilardi M.G., Cury R.G., Barbosa E.R., Fuentes R., Teixeira M.J., Fonoff E.T. (2016). Spinal cord stimulation for Parkinson’s disease: A systematic review. Neurosurg. Rev..

[23] Ahmed S., Yearwood T., De R.D., Vanneste S. (2018). Burst and high frequency stimulation: Underlying mechanism of action. Expert Rev. Med. Devices.

[24] Courtney P., Espinet A., Mitchell B., Russo M., Muir A., Verrills P., Davis K. (2015). Improved Pain Relief with Burst Spinal Cord Stimulation for Two Weeks in Patients Using Tonic Stimulation: Results From a Small Clinical Study. Neuromodulation.

[25] De Vos C.C., Bom M.J., Vanneste S., Lenders M.W., De R.D. (2014). Burst spinal cord stimulation evaluated in patients with failed back surgery syndrome and painful diabetic neuropathy. Neuromodulation.

[26] De Ridder D., van der Loo E., Van der Kelen K., Menovsky T., Van de Heyning P., Moller A. (2007). Do tonic and burst TMS modulate the lemniscal and extralemniscal system differentially?. Int. J. Med. Sci..

[27] De Ridder D., Vanneste S., Plazier M., van der Loo E., Menovsky T. (2010). Burst spinal cord stimulation: Toward paresthesia-free pain suppression. Neurosurgery.

[28] De Ridder D., Plazier M., Kamerling N., Menovsky T., Vanneste S. (2013). Burst spinal cord stimulation for limb and back pain. World Neurosurg..

[29] De Ridder D., Lenders M.W., De Vos C.C., Dijkstra-Scholten C., Wolters R., Vancamp T., Van L.P., Van H.T., Vanneste S. (2015). A 2-center comparative study on tonic versus burst spinal cord stimulation: Amount of responders and amount of pain suppression. Clin. J. Pain.

[30] De Ridder D., Perera S., Vanneste S. (2017). Are 10 kHz Stimulation and Burst Stimulation Fundamentally the Same?. Neuromodulation.

[31] Deer T.R., Campos L.W., Pope J.E. (2017). Evaluation of Abbott’s BurstDR stimulation device for the treatment of chronic pain. Expert Rev. Med. Devices.

[32] Meuwissen K.P.V., Gu J.W., Zhang T.C., Joosten E.A.J. (2018). Conventional-SCS vs. Burst-SCS and the Behavioral Effect on Mechanical Hypersensitivity in a Rat Model of Chronic Neuropathic Pain: Effect of Amplitude. Neuromodulation.

[33] De Ridder D., Vancamp T., Lenders M.W., De Vos C.C., Vanneste S. (2015). Is preoperative pain duration important in spinal cord stimulation? A comparison between tonic and burst stimulation. Neuromodulation.

[34] Schu S., Slotty P.J., Bara G., von K.M., Edgar D., Vesper J. (2014). A prospective, randomised, double-blind, placebo-controlled study to examine the effectiveness of burst spinal cord stimulation patterns for the treatment of failed back surgery syndrome. Neuromodulation.

[35] Mazzone P., Paoloni M., Mangone M., Santilli V., Insola A., Fini M., Scarnati E. (2014). Unilateral deep brain stimulation of the pedunculopontine tegmental nucleus in idiopathic Parkinson’s disease: Effects on gait initiation and performance. Gait Posture.

[36] Mazzone P., Vitale F., Capozzo A., Viselli F., Scarnati E., Krames E., Hunter Peckham P., Rezai A. (2018). Deep brain stimulation of the pedunculopontine tegmental nucleus improves static balance in Parkinson’s disease. Neuromodulation 2nd.

[37] Benis D., David O., Piallat B., Kibleur A., Goetz L., Bhattacharjee M., Fraix V., Seigneuret E., Krack P., Chabardes S. (2016). Response inhibition rapidly increases single-neuron responses in the subthalamic nucleus of patients with Parkinson’s disease. Cortex.

[38] Brunamonti E., Ferraina S., Pare M. (2012). Controlled movement processing: Evidence for a common inhibitory control of finger, wrist, and arm movements. Neuroscience.

[39] Mione V., Canterini S., Brunamonti E., Pani P., Donno F., Fiorenza M.T., Ferraina S. (2015). Both the COMT Val158Met single-nucleotide polymorphism and sex-dependent differences influence response inhibition. Front. Behav. Neurosci..

[40] Olivito G., Brunamonti E., Clausi S., Pani P., Chiricozzi F.R., Giamundo M., Molinari M., Leggio M., Ferraina S. (2017). Atrophic degeneration of cerebellum impairs both the reactive and the proactive control of movement in the stop signal paradigm. Exp. Brain Res..

[41] Battaglia-Mayer A., Buiatti T., Caminiti R., Ferraina S., Lacquaniti F., Shallice T. (2014). Correction and suppression of reaching movements in the cerebral cortex: Physiological and neuropsychological aspects. Neurosci. Biobehav. Rev..

[42] Rocchi C., Pierantozzi M., Galati S., Chiaravalloti A., Pisani V., Prosperetti C., Lauretti B., Stampanoni B.M., Olivola E., Schillaci O. (2015). Autonomic Function Tests and MIBG in Parkinson’s Disease: Correlation to Disease Duration and Motor Symptoms. CNS Neurosci. Ther..

[43] Rocchi C., Pierantozzi M., Pisani V., Marfia G.A., Di G.A., Stanzione P., Bernardi G., Stefani A. (2012). The impact of rotigotine on cardiovascular autonomic function in early Parkinson’s disease. Eur. Neurol..

[44] Pstras L., Thomaseth K., Waniewski J., Balzani I., Bellavere F. (2016). The Valsalva manoeuvre: Physiology and clinical examples. Acta Physiol. (Oxf.).

[45] Verbruggen F., Logan G.D. (2009). Models of response inhibition in the stop-signal and stop-change paradigms. Neurosci. Biobehav. Rev..

[46] Nishioka K., Nakajima M. (2015). Beneficial therapeutic effects of spinal cord stimulation in advanced cases of Parkinson’s disease with intractable chronic pain: A case series. Neuromodulation.

[47] Santana M.B., Halje P., Simplicio H., Richter U., Freire M.A.M., Petersson P., Fuentes R., Nicolelis M.A.L. (2014). Spinal cord stimulation alleviates motor deficits in a primate model of Parkinson disease. Neuron.

[48] Crosby N.D., Weisshaar C.L., Smith J.R., Zeeman M.E., Goodman-Keiser M.D., Winkelstein B.A. (2015). Burst and Tonic Spinal Cord Stimulation Differentially Activate GABAergic Mechanisms to Attenuate Pain in a Rat Model of Cervical Radiculopathy. IEEE Trans. Biomed. Eng..

[49] Ahmed Z., Wieraszko A. (2012). Trans-spinal direct current enhances corticospinal output and stimulation-evoked release of glutamate analog, D-2,3-(3)H-aspartic acid. J. Appl. Physiol. (1985).

[50] Garcia-Rill E., Luster B., D’Onofrio S., Mahaffey S., Bisagno V., Urbano F.J. (2015). Pedunculopontine arousal system physiology—Deep brain stimulation (DBS). Sleep Sci..

[51] Garcia-Rill E., Luster B., D’Onofrio S., Mahaffey S., Bisagno V., Urbano F.J. (2016). Implications of gamma band activity in the pedunculopontine nucleus. J. Neural Transm. (Vienna).

[52] Mazzone P., Vilela F.O., Viselli F., Insola A., Sposato S., Vitale F., Scarnati E. (2016). Our first decade of experience in deep brain stimulation of the brainstem: Elucidating the mechanism of action of stimulation of the ventrolateral pontine tegmentum. J. Neural Transm. (Vienna).

[53] Garcia-Rill E., Mahaffey S., Hyde J.R., Urbano F.J. (2018). Bottom-up gamma maintenance in various disorders. Neurobiol. Dis..

[54] Mazzone P., Pisani R., Pizio N., Arrigo A., Nobili F. (1994). Cerebral blood flow and somatosensory evoked response changes induced by spinal cord stimulation: Preliminary follow-up observations. Stereotact. Funct. Neurosurg..

[55] Mazzone P., Pisani R., Nobili F., Arrigo A., Gambaro M., Rodriguez G. (1995). Assessment of regional cerebral blood flow during spinal cord stimulation in humans. Stereotact. Funct. Neurosurg..

[56] Mazzone P., Rodriguez G., Arrigo A., Nobili F., Pisani R., Rosadini G. (1996). Cerebral haemodynamic changes induced by spinal cord stimulation in man. Ital. J. Neurol. Sci..

[57] Rasche D., Siebert S., Stippich C., Kress B., Nennig E., Sartor K., Tronnier V.M. (2005). Spinal cord stimulation in Failed-Back-Surgery-Syndrome. Preliminary study for the evaluation of therapy by functional magnetic resonance imaging (fMRI). Schmerz.

[58] Si J., Dang Y., Zhang Y., Li Y., Zhang W., Yang Y., Cui Y., Lou X., He J., Jiang T. (2018). Spinal Cord Stimulation Frequency Influences the Hemodynamic Response in Patients with Disorders of Consciousness. Neurosci. Bull..

[59] Nambu A., Tokuno H., Takada M. (2002). Functional significance of the cortico-subthalamo-pallidal ‘hyperdirect’ pathway. Neurosci. Res..

[60] Nambu A. (2005). A new approach to understand the pathophysiology of Parkinson’s disease. J. Neurol..

[61] Canolty R.T., Edwards E., Dalal S.S., Soltani M., Nagarajan S.S., Kirsch H.E., Berger M.S., Barbaro N.M., Knight R.T. (2006). High gamma power is phase-locked to theta oscillations in human neocortex. Science.

[62] Liu C.C., Chien J.H., Kim J.H., Chuang Y.F., Cheng D.T., Anderson W.S., Lenz F.A. (2015). Cross-frequency coupling in deep brain structures upon processing the painful sensory inputs. Neuroscience.

[63] Voloh B., Valiante T.A., Everling S., Womelsdorf T. (2015). Theta-gamma coordination between anterior cingulate and prefrontal cortex indexes correct attention shifts. Proc. Natl. Acad. Sci. USA.

[64] De Ridder D., van der Loo E., Van der Kelen K., Menovsky T., Van de Heyning P., Moller A. (2007). Theta, alpha and beta burst transcranial magnetic stimulation: Brain modulation in tinnitus. Int. J. Med. Sci..

[65] Llinas R.R., Ribary U., Jeanmonod D., Kronberg E., Mitra P.P. (1999). Thalamocortical dysrhythmia: A neurological and neuropsychiatric syndrome characterized by magnetoencephalography. Proc. Natl. Acad. Sci. USA.

[66] Von Stein A., Sarnthein J. (2000). Different frequencies for different scales of cortical integration: From local gamma to long range alpha/theta synchronization. Int. J. Psychophysiol..

[67] Ding X., Mountain D.J., Subramanian V., Singh K., Williams C.A. (2007). The effect of high cervical spinal cord stimulation on the expression of SP, NK-1 and TRPV1 mRNAs during cardiac ischemia in rat. Neurosci. Lett..

[68] Simpson R.K., Robertson C.S., Goodman J.C., Halter J.A. (1991). Recovery of amino acid neurotransmitters from the spinal cord during posterior epidural stimulation: A preliminary study. J. Am. Paraplegia Soc..

[69] Ultenius C., Song Z., Lin P., Meyerson B.A., Linderoth B. (2013). Spinal GABAergic mechanisms in the effects of spinal cord stimulation in a rodent model of neuropathic pain: Is GABA synthesis involved?. Neuromodulation.

